# Danggui-Shaoyao-San modulates sphingolipid metabolism to promote oligodendrocyte differentiation and maturation in vascular dementia rats

**DOI:** 10.1186/s13020-025-01284-x

**Published:** 2026-01-07

**Authors:** Yue Su, Yuying Zhong, Ningning Yuan, Xiang Li, Ying Xu, Hui Yang, Mengmeng Huang, Yafeng Zhang, Xiaolan Cheng

**Affiliations:** 1 School of Integrated Chinese and Western Medicine, Nanjing University of Chinese Medicine, Nanjing, 210023 China; 2 School of Pharmacy, Nanjing University of Chinese Medicine, Nanjing, 210023 China; 3 Jiangsu CM Clinical Innovation Center of Degenerative Bone & Joint Disease, Wuxi TCM Hospital Affiliated to Nanjing University of Chinese Medicine, Wuxi, 214071 China

**Keywords:** Vascular dementia, Danggui Shaoyao San, Myelin regeneration, Oligodendrocyte precursor cells, Sphingolipid metabolism, SPHK2

## Abstract

**Objective:**

Vascular dementia (VaD) is a neurodegenerative disease primarily characterized by white matter injury and myelin degeneration, and currently, there is a lack of effective treatment options. This study aims to investigate the effects of the traditional Chinese medicine formula Danggui Shaoyao San (DSS) on cognitive function and myelin repair in VaD rats and to elucidate its underlying mechanisms.

**Methods:**

The VaD rat model was established using the bilateral common carotid artery ligation (2VO) method. The effects of DSS on cognitive function, myelin regeneration, sphingolipid metabolism, and SPHK2/S1P/S1PR5 pathway was conducted using behavioral tests, histological staining, Western blot, lipidomics, qPCR, immunofluorescence, LC–MS/MS, and 16S rRNA sequencing. Besides, molecular docking and molecular dynamics simulation were carried out.

**Results:**

DSS treatment significantly improved learning and memory abilities in VaD rats, reduced structural damage in the hippocampus and white matter, and promoted the differentiation of oligodendrocyte precursor cells (OPCs) into mature oligodendrocytes (OLs). Lipidomics and molecular biological experiments indicated that DSS activated the SPHK2/S1P/S1PR5 pathway, ameliorated sphingolipid metabolic disorders and increased S1P levels, thereby promoting myelin repair. The specific SPHK2 inhibitor ABC294640 significantly weakened the neuroprotective effects of DSS, further confirming the central role of SPHK2/S1P/S1PR5 pathway. Antibiotic depletion experiments confirmed that the gut microbiota was not a key mediator of the therapeutic effects of DSS. Finally, molecular docking and molecular dynamics simulations indicated that the DSS components Albiflorin and Gallic acid form tighter and more stable interactions with SPHK2.

**Conclusion:**

DSS improved VaD cognitive impairment by modulating sphingolipid metabolism and promote myelin regeneration via activating the SPHK2/S1P/S1PR5 signaling pathway. This study provides important experimental evidence for the clinical application of DSS in VaD.

## Introduction

Vascular Dementia (VaD) is a cognitive impairment caused by cerebrovascular lesions and is the second most common type of dementia in the elderly after Alzheimer's Disease (AD), accounting for 15% to 20% of all dementia cases [1–3]. In terms of clinical manifestations, patients with VaD typically experience memory loss, impaired speech, and executive dysfunction, which significantly reduce their quality of life [4, 5]. The pathogenesis of VaD is complex and involves several key components, including neuronal apoptosis, neuroinflammation, cholinergic system dysfunction, and oxidative stress [6]. Among them, white matter damage and demyelination are the important pathological basis of VaD [7]. The integrity of myelin sheaths is essential for maintaining efficient nerve signaling, and myelin damage is often accompanied by cognitive decline [8]. Therefore, promoting myelin regeneration has become an important strategy in the current clinical treatment of VaD [9].

Myelin is primarily formed by oligodendrocytes (OLs), which differentiate from oligodendrocyte precursor cells (OPCs) [10, 11]. Under pathological conditions such as cerebral ischemia or other neurological injuries, the proliferation of OPCs can be activated, but their differentiation into mature OLs is often impeded, leading to the failure of myelin regeneration [12]. Therefore, promoting the differentiation of OPCs is important for restoring myelin structure [13, 14]. Recent studies have highlighted that lipid metabolism, especially sphingolipid metabolism, is closely associated with OPC proliferation and differentiation [15]. Sphingolipids are important components of cell membranes, and the dynamic balance between their metabolites ceramide (Cer) and sphingosine-1-phosphate (S1P) is pivotal for cell survival, differentiation, and nerve regeneration [16, 17]. Specifically, Cer exerts pro-apoptotic effect, while S1P is neuroprotective and promotes cell survival [18]. In animal models of vascular dementia (VaD), Cer accumulates significantly whereas S1P levels are markedly reduced, suggesting that an imbalance in sphingolipid metabolism is closely related to the demyelination process of VaD [19]. Sphingosine kinase 2 (SPHK2), a key enzyme that catalyzes the conversion of sphingosine (Sph) to S1P, is mainly located in oligodendrocytes of the central nervous system [20]. S1P generated by SPHK2 acts through its receptor S1PR5, which in turn activates the transcription factor SOX10, thereby promoting OPC differentiation and myelin formation. Thus, the SPHK2/S1P/SOX10 pathway is considered an important regulatory axis for myelin regeneration. However, there is a lack of systematic studies on the specific role of this pathway in VaD-related demyelination.

Danggui-Shaoyao San (DSS) is a classic blood-regulating formula from the ancient Chinese medical text *The Essentials of the Golden Chamber*. It is composed of *Angelica Sinensis* (Oliv.) Diels., Radix Paeoniae Alba, *Ligusticum chuanxiong* Hort, *Atractyolodes macrocephala* Koidz., *Poria cocos* (Schw.) Wolf., and *Alisma orientale* (Sam.) Juzep. DSS is renowned for its therapeutic efficacy in nourishing the blood, softening the liver, invigorating the spleen, and dispelling dampness [21, 22]. Both clinical studies and animal experiments have confirmed the ameliorative effect of DSS on vascular dementia (VaD) [23, 24]. In addition, modern pharmacological studies have identified a variety of active ingredients in DSS, which exhibit antioxidant, anti-inflammatory, immunity regulating and neuroprotective effects. These components are able to significantly dilate peripheral blood vessels, improve microcirculation, and promote cerebral metabolism activation [25]. However, the effects of DSS on myelin regeneration in VaD, as well as the underlying mechanisms, remain unclear.

The present study was designed to establish a VaD rat model by permanent ligation of bilateral common carotid arteries (2VO), with the aim of evaluating the effects of DSS on spatial learning and memory abilities, myelin structure, OPCs differentiation and sphingolipid metabolism, and exploring the specific molecular mechanisms of DSS in the treatment of VaD.

## Experimental materials and methods

### Preparation of DSS extract

DSS extract was prepared by combining *Angelica Sinensis*(Oliv.) Diels., Radix Paeoniae Alba, *Atractyolodes macrocephala* Koidz., *Alisma orientale* (Sam.) Juzep., *Ligusticum chuanxiong* Hort, *Poria cocos* (Schw.) Wolf. in the ratio of 3:16:4:8:3:4. The mixture was soaked for 1 h, and then refluxed for two times (8:1, *v/w* and 6:1, *v/w*), 2 h for each. The filtrate was combined and evaporated to 2 g/mL of raw herbs by rotary evaporator. The concentrated extract was stored at − 80℃ for subsequent drug administration and was freshly prepared for each use.

### Animal and experimental design

Male SPF-grade SD rats, weighing 280 ± 30 g, were used in this study. The rats were obtained from Shanghai Slaughter Laboratory Animal Center Company, and housed in a clean-grade environment with a temperature of 20–26 °C and relative humidity of 40–70%, under a 12-h day/night cycle. All animal experiments were approved by the Ethics Committee for Animal Experiments of Nanjing University of Chinese Medicine (ethics approval number: NO.202211A029, NO. 202304A030).

#### Animal modeling and experimental grouping for pharmacodynamic studies

VaD rat model was prepared by bilateral common carotid artery ligation (2VO) method. The rats were anesthetized and fixed. After disinfection, an incision was made in the neck skin, and the muscle tissues were bluntly dissected to expose the bilateral common carotid arteries. The proximal and distal ends of the carotid arteries were carefully separated, ligated and sutured with 4.0 suture. In the sham group, the common carotid arteries were separated only but not ligated. The rats were randomly divided into 5 groups (n = 10): sham group, model group, L-DSS (5 g/kg) group, H-DSS (10 g/kg) group and Nimodipine (NIM) group. Rats of sham and model groups were given an equal volume of distilled water by gavage. Rats in L-DSS group, H-DSS group and NIM group were respectively administered 5 g/kg of DSS, 10 g/kg of DSS or 9.45 mg/kg of nimodipine by gavage for 4 weeks.

#### Animal modeling and intervention protocols for mechanism research

After the VaD rat model was established using the 2VO method, experimental animals were randomly divided into 6 groups (n = 10 per group) and treated as follows:

- Sham group: rats were administered with an equal volume of saline by gavage.
- Model group: rats were administered with an equal volume of saline by gavage.
- DSS group: rats were administered with 10 g/kg DSS daily by gavage.
- Antibiotic compound group (Ant): rats were provided free access to water containing neomycin (1 g/L), vancomycin (50 mg/L) and metronidazole (200 mg/L).
- Antibiotic compound + DSS group (AD): rats were provided free access to the antibiotic compound water and administered 10 g/kg DSS by gavage.
- ABC294640 (SphK2 inhibitor) + DSS group (ABC): rats were administered 10 g/kg DSS daily by gavage and 10 mg/kg ABC294640 via intraperitoneal injection every other day.

Drug treatment commenced 1 week after model establishment and continued for 4 weeks, with behavioral testing conducted at the end of the treatment period.

### Behavioral tests

#### New objects recognition

The novel object recognition test consists of a training period and a test period. During the training period, two objects of the same size, A (a cylinder) and B (a cone), are placed parallel to the two corners of the experimental box. The rat was placed in the center of the box, equidistant from both objects, and allowed to explore freely for 5 min. Exploration is considered successful when the rat’s nose is within 2 cm of an object.

One hour after the training period, object B was replaced with a new object C (a rectangular object). The rat was placed back in the center of the box and allowed to explore freely for 5 min. The exploration time for the new object C and the familiar object A is recorded. This procedure is repeated after 24 h. The rats' ability to recognize new and familiar objects is assessed by calculating the discrimination index (DI) = t_C_/(t_C_ + t_A_) × 100%, where t_C_ is the exploration time for the new object C and t_A_ is the exploration time for the familiar object A.

#### Morris water maze

Morris water maze was used to detect the learning and memory abilities of rats at the end of drug administration. The pool was divided into four quadrants and the escape platform was placed in the third (target) quadrant. On day 1, rats form all groups were placed in the pool for environmental familiarization. From days 2 to 5, rats underwent daily training trials. Rats were placed facing the wall in quadrant 1, and the escape latency (time taken to find the platform) was recorded. Rats remained on the platform for 30 s. If a rat failed to find the platform within 60 s, it was guided there and the escape latency was recorded as 60 s. Each day, an additional training trial was conducted starting from quadrant 2 using the same procedure. On day 6, the platform was removed. Rats were placed in quadrant 1 and allowed to swim freely for 60 s. The following were recorded to evaluate learning and memory: time spent in the target quadrant, escape latency (to the former platform location), and the number of times the rat crossed the original platform position.

### Histologic and ultrastructural examination

#### Luxol fast blue (LFB) staining for myelin damage

To evaluate the integrity of myelin in the white matter region of rats, the corpus callosum region was selected for LFB staining. Three coronal sections of brain tissue per rat group were deparaffinized, rehydrated, and incubated in preheated LFB staining solution at 60 °C for 3 h. Subsequently, the sections were processed by ethanol and differentiation solution for differentiation, dehydration, transparency and sealing. Myelin staining density and vacuolation were observed under the microscope, and full-section images were acquired for semi-quantitative analysis of myelin loss severity.

#### Transmission electron microscopy for myelin ultrastructural alterations

To further analyze myelin ultrastructure, transmission electron microscopy (TEM) was used to examine the corpus callosum and white matter region. Fresh brain tissue were primarily fixed in glutaraldehyde, post-fixed in 1% osmium tetroxide (aqueous), and dehydrated through an acetone gradient. After that, tissues were then embedded using Epon-812. Ultrathin sections with a thickness of 60 nm were prepared and contrast stained with uranyl acetate and lead citrate. Finally, images were taken under a transmission electron microscope to analyze the thickness of myelin sheaths, compaction density, and axon-myelin interactions.

### Western blot analysis

Total protein of white matter was extracted using protein lysate and protein concentration was determined using a BCA assay kit (Beyotime, China). Subsequently, equal amount of protein from each sample was resolved by SDS-PAGE and transferred to a PVDF membrane (Millipore, USA). The membrane was blocked using 5% skimmed milk at room temperature powder for 1 h. Next, the membrane was incubated separately with primary antibodies (MBP, MAG, p38MAPK, Sox10, Myrf, SPHK2, S1PR5, diluted at a ratio of 1:1000) at 4 °C overnight, and then incubated with HRP-labeled secondary antibody for 1 h at room temperature. Protein bands were detected using ECL luminescent solution and visualized with an imaging system. The target bands were analyzed in grayscale using Image J software, and normalized to GAPDH as the internal reference protein.

### Real-time quantitative PCR (RT-qPCR) analysis

Total RNA of white matter was extracted using Trizol reagent (Invitrogen, USA), and then reverse transcribed to cDNA. RT-qPCR was performed using SYBR Green PCR Master Mix. The initial denaturation set at 95 °C for 3 min, followed by 40 cycles of denaturation at 95 °C for 10 s and annealing/extension at 60 °C for 30 s. The relative expression was calculated using the 2^−ΔΔCt^ method with GAPDH as the internal reference gene. All samples were subjected to three replicate experiments. The PCR primers used in the study are listed in Table 1.

**Table 1:** Target primer sequences

Gene	Forward Primer (5’−3’)		Reverse Primer (5’−3’)
MBP		AGGCGTAGAGGAACTATGGGT	TCACCACTGTCCAATCAGGG
MAG		TCAACAGTCCCTACCCCAAG	GAGAAGCAGGGTGCAGTTTC
Sox10		GACCCTATTATGGCCACGCA	GCCCCTCTAAGGTCGGGATA
Myrf		CCTGTGTCCGTGGTACTGTG	TCACACAGGCGGTAGAAGTG
Sphk1		GATGCATGAGGTGGTGAATG	AACAGCAGTGTGCAGTTGAT
Sphk2		GAAGGCATTGTCACTGTGTC	GCAGAGAAGAAGCGAGCAGT
S1pr1		GGATCGCGCGGTGTAGAC	GCTAGAGGGCGAGGTTGAG
S1pr3		CCTCATCACCACCATCCTCT	TGGAGTAGAGGGGCAAGATG
S1pr5		CCAAGGCCTATGTGCTCTTC	GTTGGAGGAGTCTTGGTTGC
Gapdh		TCCAGTATGACTCTACCCACG	CACGACATACTCAGCACCAG

### Immunofluorescence staining

Brain tissues were fixed in 4% paraformaldehyde solution for 24 h, cryoprotected in 30% sucrose, and sectioned coronally at 20 μm. After washing with PBS, sections were blocked with 5% BSA for 30 min at room temperature, and incubated overnight at 4 °C with primary antibodies against MBP, Olig2, NG2 (1:200 dilution). The following day, sections were incubated with secondary antibodies (Alexa Fluor 488 or 594) for 1 h at room temperature, followed by re-staining of the nuclei with DAPI. Sections were observed and images were acquired using a confocal fluorescence microscope (Zeiss LSM800, Germany).

### Enzyme-linked immunosorbent assay (ELISA)

The ELISA kit was used to detect inflammatory factors or metabolic indexes (e.g. IL-1β, TNF-α, S1P) in rat serum or tissue homogenate. The absorbance was measured at 450 nm.

### Sphingolipid metabolomics analysis (LC–MS/MS)

Sphingolipid metabolomic profiling was performed in brain tissues were examined using chromatography-tandem mass spectrometry (LC–MS/MS). First, the tissue samples were subjected to lipid extraction using a methanol: chloroform (2:1, v/v) mixture. The extracted lipid samples were further analyzed using an LC–MS/MS (SCIEX TripleQuad 6500 + MS/MS) platform. Chromatographic separation employed a reversed-phase C_18_ column (2.1 × 100 mm, 1.7 μm) with a 0.4 mL/min gradient of (A) 0.1% formic acid in water and (B) 0.1% formic acid in acetonitrile: isopropanol (60:40). The mass spectrometry data were internal standardized for peak area and then imported into matlab software for analysis. Differentially abundant sphingolipids were screened based on the orthogonal partial least squares-discriminant analysis (OPLS-DA) analysis with thresholds of variable importance projection (VIP) value > 1 and P value < 0.05.

### 16S rRNA sequencing analysis of intestinal flora

Rat fecal samples were collected, and total genomic DNA of rat intestinal flora was extracted using MagPure Soil DNA LQ Kit. The V4-V5 region of the bacterial 16S rRNA gene was amplified by PCR using Takara Ex Taq High Fidelity Enzyme, and then sequenced by Illumina NovaSeq 6000. The sequencing data were denoised, spliced and clustered by QIIME2 to analyze the composition of bacterial colonies, α and β diversity analysis and species abundance changes. Principal coordinate analysis (PCoA) was performed to assess the β-diversity of the samples using the unweighted Unifrac distance matrix calculated by R.

### In-depth analysis of the interaction of DSS active ingredients with SPHK2 by molecular docking and molecular dynamics (MD) simulation

In this study, the molecular docking technique was used to perform an in-depth analysis of the interactions of the major active ingredients of DSS with SPHK2, whose three-dimensional structural data were derived from the AlphaFold Protein Structure Database [26]. The molecular structures of ligustrazine, albiflorin, ligustilide, ferulic acid and gallic acid were obtained from PubChem database. The molecular docking experiments were carried out by using AutoDockTools 1.5.6 software. PyMOL software was applied to visualize the docking results. For molecular dynamics simulations, the Gromacs2022 program was employed, combined with the GAFF force field for small molecules, the AMBER14SB force field for proteins, and the TIP3P water model to construct the complex simulation system. These simulations was carried out at constant temperature and pressure as well as under periodic boundary conditions. The LINCS algorithm was used to constrain hydrogen bonds, and the PME method was applied to calculate electrostatic interactions. After 100 ps of equilibration simulations, 100 ns of MD simulations were carried out, with conformations saved every 10 ps. After the simulations were completed, the trajectories were analyzed using VMD and PyMOL, and the MMPBSA binding free energy analysis between the protein and the small-molecule ligand was performed using the g_mmpbsa program.

### Microscale thermophoresis (MST)

MST measurements were conducted using a Monolith NT.115 (NanoTemper) to assess the binding affinities of four compounds such as ligustrazine, ferulic acid, albiflorin, and gallic acid, to recombinant SPHK2 protein. Recombinant SPHK2 protein was purchased from Abcam (Anti-SPHK2 antibody [EPR29189-33]), and the concentration of SPHK2 protein was quantified by measuring the total fluorescence of the labeled SPHK2 using a Pico-RED-based labeling system. A titration series of Ligustrazine, Ferulic acid, Albiflorin, and Gallic acid concentrations (1 mM–1 μM) was prepared. Equal volumes of each compound and the labeled SPHK2 protein solution were combined and incubated to reach binding equilibrium.

The samples were then into MST NT.115 standard glass capillaries, and the binding affinity of each compound for SPHK2 was quantified. The dissociation constant (Kd) for each interaction was analyzed using MO.affinity software (Analysis v2.3). A lower Kd value indicates a stronger binding interaction. The results demonstrate that ligustrazine, ferulic acid, albiflorin, and gallic acid all bind effectively to the SPHK2 protein.

### Statistical analysis

The experimental data were analyzed using GraphPad Prism 9.0 software. Results are expressed as means ± SEM. For comparisons between two groups, the unpaired t-test was employed, provided the data met the assumptions of normality and homogeneity of variances, which were assessed using the Shapiro–Wilk test and Levene’s test, respectively.

For comparisons involving three or more groups, one-way analysis of variance (ANOVA) was conducted, followed by Tukey’s post-hoc test for multiple comparisons. Prior to performing one-way ANOVA, the data were tested for normality using the Shapiro–Wilk test and for homogeneity of variances using Levene’s test. The results confirmed that the data met the assumptions of normality and homogeneity of variances, making the use of one-way ANOVA appropriate. Statistical significance was defined as *P* < 0.05.

## Results

### DSS improved learning memory function of VaD rats

**Fig. 1:**
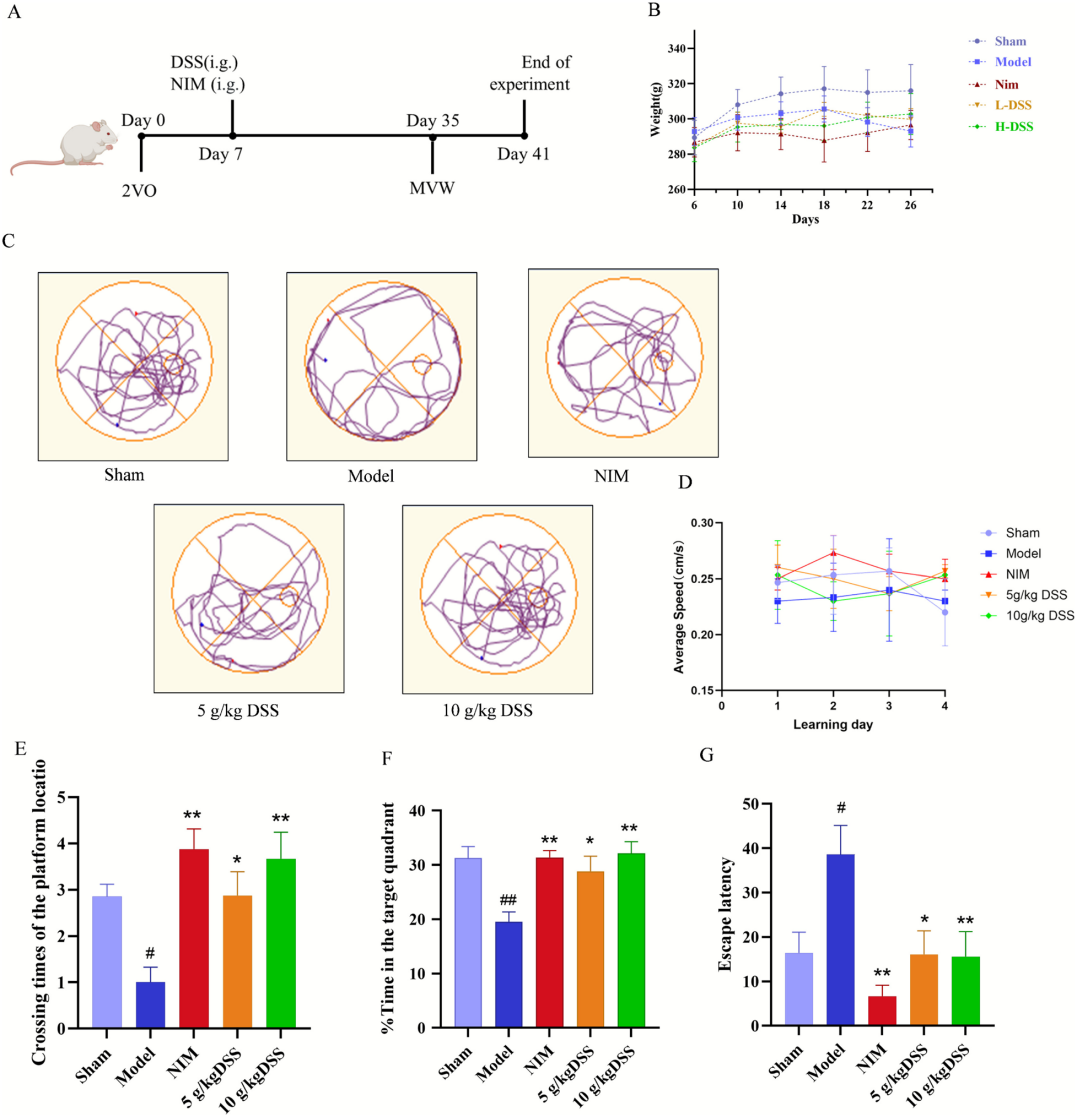
DSS improved learning memory function of VaD rats. **A** Flowchart of the experimental procedure. **B** Body weight changes in rats throughout the experimental period. **C** Path traces during the morris water maze test. **D** Average swimming speed in the Morris water maze experiment (**E**) Times of platform crossings in the Morris water maze test. **F** Time spent in the target quadrant during the Morris water maze test. **G** Escape latency in the Morris water maze test

Cognitive function in VaD rats was evaluated using the Morris water maze test. The flowchart of the experimental procedure was exhibited in Fig. 1A. As shown in Fig. 1B, DSS had little effect on the body weight of rats. During the place navigation phase, rats in the model group exhibited decreased number of platform crossings, shortened the time spent in the target quadrant wa well as prolonged escape latency versus sham controls (*P* < 0.05), indicating spatial learning impairment (Fig. 1C-G). However, DSS dose-dependently increased the number of platform crossings, prolonged the time spent in the target quadrant, and shortened the escape latency of VaD rats, with high-dose DSS (H-DSS) demonstrating the most pronounced effect (*P* < 0.05). The average swimming speed did not differ significantly between groups. Thus, the observed cognitive deficits in VaD rats are attributable to impaired spatial learning and memory rather than to motor dysfunction (Fig. 1D). The above results indicated that DSS could effectively reverse the learning and memory deficits of VaD rats.

### DSS improved the structural damage of hippocampal and white matter tissue

**Fig. 2:**
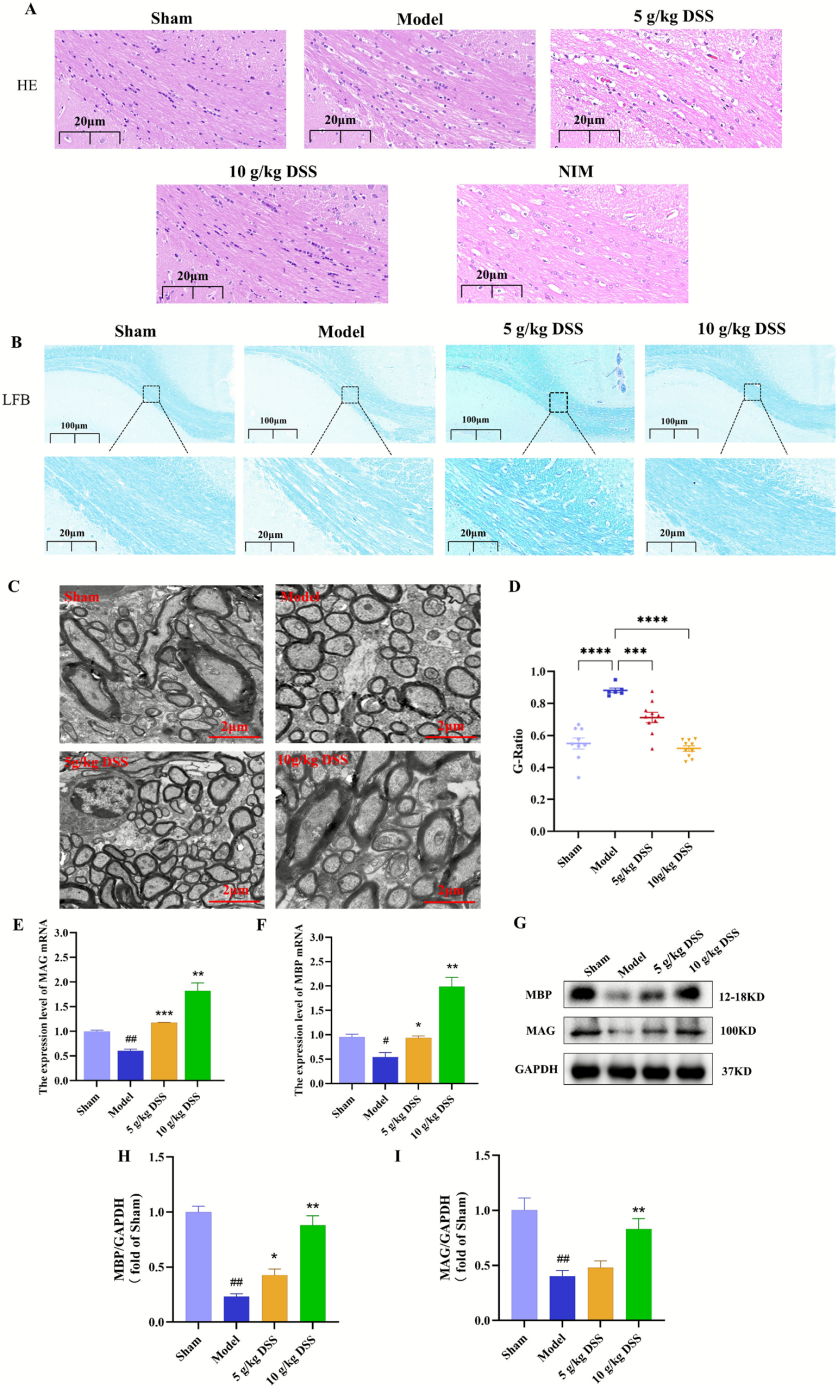
DSS improved the structural damage of hippocampal and white matter tissue. **A** Hematoxylin and eosin (HE) staining of the hippocampal CA1 region. **B** Luxol Fast Blue (LFB) staining of white matter. **C** Transmission electron microscopy (TEM) images displaying the ultrastructure of myelin sheaths. **D** G-ratio analysis of myelin sheaths. **E**–**F** Quantification of MAG and MBP expression in white matter of rats by RT-qPCR. **G** Western blot analysis of MAG and MBP expression in white matter of rats. **H**-**I** Quantification of MAG and MBP expression from Western blot data

As exhibited in Fig. 2A, HE staining results showed that neurons in CA1 region of the hippocampus in sham group were neatly arranged and structurally intact, while neurons in Model group were disorganized, with shrunken cytoplasm, deepened staining, and obvious vacuolization and cell loss in the white matter area. However, the H-DSS and NIM groups showed well-preserved neuronal morphology, with neurons appearing intact and well-arranged. L-DSS group exhibited slight improvement in neuronal injury compared to the model group.

LFB staining highlighted the extent of demyelination in the white matter region (Fig. 2B). The Model group displayed obvious demyelination, characterized by lighter staining and sparse myelin structure. In contrast, after DSS intervention, the staining intensity increased, and the myelin structure appeared more robust. This finding suggests that DSS can promote remyelination in the damaged white matter. TEM observation further confirmed the differences in myelin structure. The myelin sheaths in the model group were thinned, loosely structured, and axons were partially exposed. In contrast, the H-DSS group exhibited thicker and more densely arranged myelin sheaths, indicating improved myelin integrity (Fig. 2C). G-ratio is a key indicator of myelin thickness, reflecting the ratio of axon diameter to the total diameter of the myelinated. A G-ratio value closer to 1 indicates more severe demyelination. As shown in Fig. 2D, the model group showed significant demyelination, with a G-ratio value close to 1 (*P* < 0.0001). In contrast, the DSS-treated group had a significantly lower G-ratio value (*P* < 0.001; *P* < 0.0001), indicating increased myelin thickness. These results further support the notion that DSS can effectively alleviate demyelination and promote myelin regeneration in the white matter region of VaD rats.

MBP and MAG are key components of myelin, which are specifically expressed in mature oligodendrocytes. As shown in Fig. 2E-I**,** the expression of MBP and MAG at both the gene and protein level was significantly decreased in the model group compared to the sham group (*P* < 0.05; *P* < 0.0001). Notably, rats treated with 10 g/kg DSS (H-DSS) exhibited significantly elevated levels of MBP and MAG in the white matter. In summary, DSS effectively alleviated hippocampal and white matter tissue structural damage in VaD rats by promoting myelin regeneration.

### DSS promoted the differentiation of oligodendrocyte precursor cells (OPCs) into mature oligodendrocytes

**Fig. 3:**
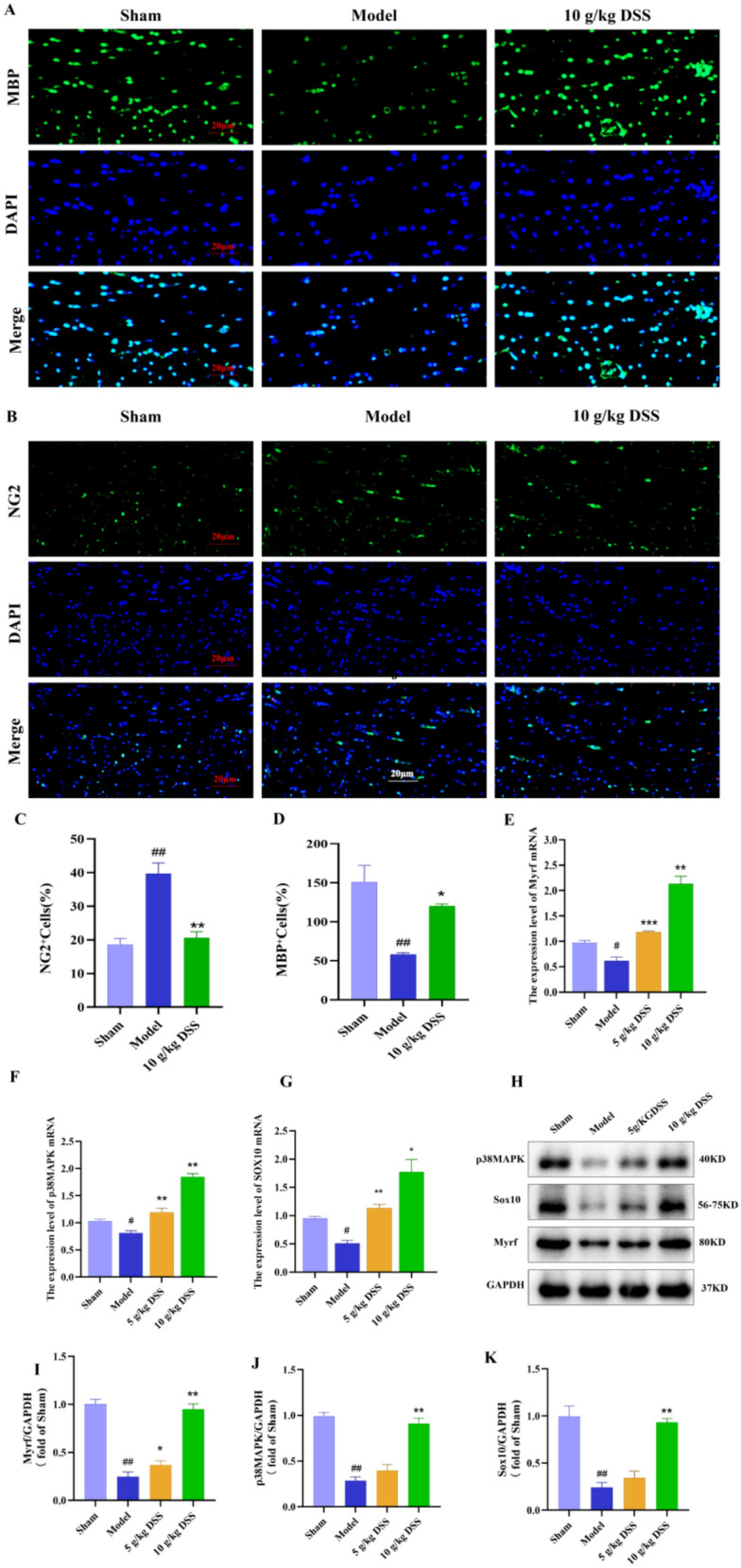
DSS promoted OPC differentiation in VaD rats. **A**–**D** Immunofluorescence staining for oligodendrocyte precursor cells (NG2) and mature oligodendrocytes (MBP). **E**–**G** Gene expression analysis of differentiation markers (p38MAPK, Sox10, Myrf) in white matter of rats by RT-qPCR. **H**–**K** Western blot analysis of p38MAPK, Sox10 and Myrf in white matter of rats

To elucidate DSS-mediated myelin regeneration mechanisms, immunofluorescence, RT-qPCR and Western blot were used to examine the expression of markers associated with OPCs and mature OLs. MBP serves as a signature protein of mature OLs. NG2, a chondroitin sulfate proteoglycan, is one of the typical markers of OPCs and is highly expressed on the cell surface. As illustrated in Fig. 3A-D, in rats of the model group, the number of mature OLs was significantly lower than that in the sham group; however, the number of OLs cells was significantly increased after treatment with DSS (*P* < 0.05). However, treatment with DSS significantly increased the number of mature OLs (*P* < 0.05). Conversely, the number of NG2 + OPCs was significantly higher in the model group compared to the sham group (*P* < 0.01), but this number was significantly reduced after DSS treatment (*P* < 0.01) (Fig. 3 A-D). As shown in Fig. 3E-G, the gene expression of transcription factors related to oligodendrocyte differentiation including p38MAPK, Sox10 and Myrf, were significantly decreased in the model group compared to the sham group (P < 0.05), and DSS treatment was able to reverse this phenomenon (*P* < 0.01, *P* < 0.001, *P* < 0.0001). WB analysis further demonstrated that DSS also significantly increased the proteins levels of the above factors (*P* < 0.0001) (Fig. 3H-K). Taken together, we found that OPCs differentiation in the white matter of VaD rats was impaired. DSS stimulated OPCs differentiation, which likely contributes to its improvement of myelin regeneration in VaD.

### DSS improved sphingolipid metabolism levels in VaD rats

**Fig. 4:**
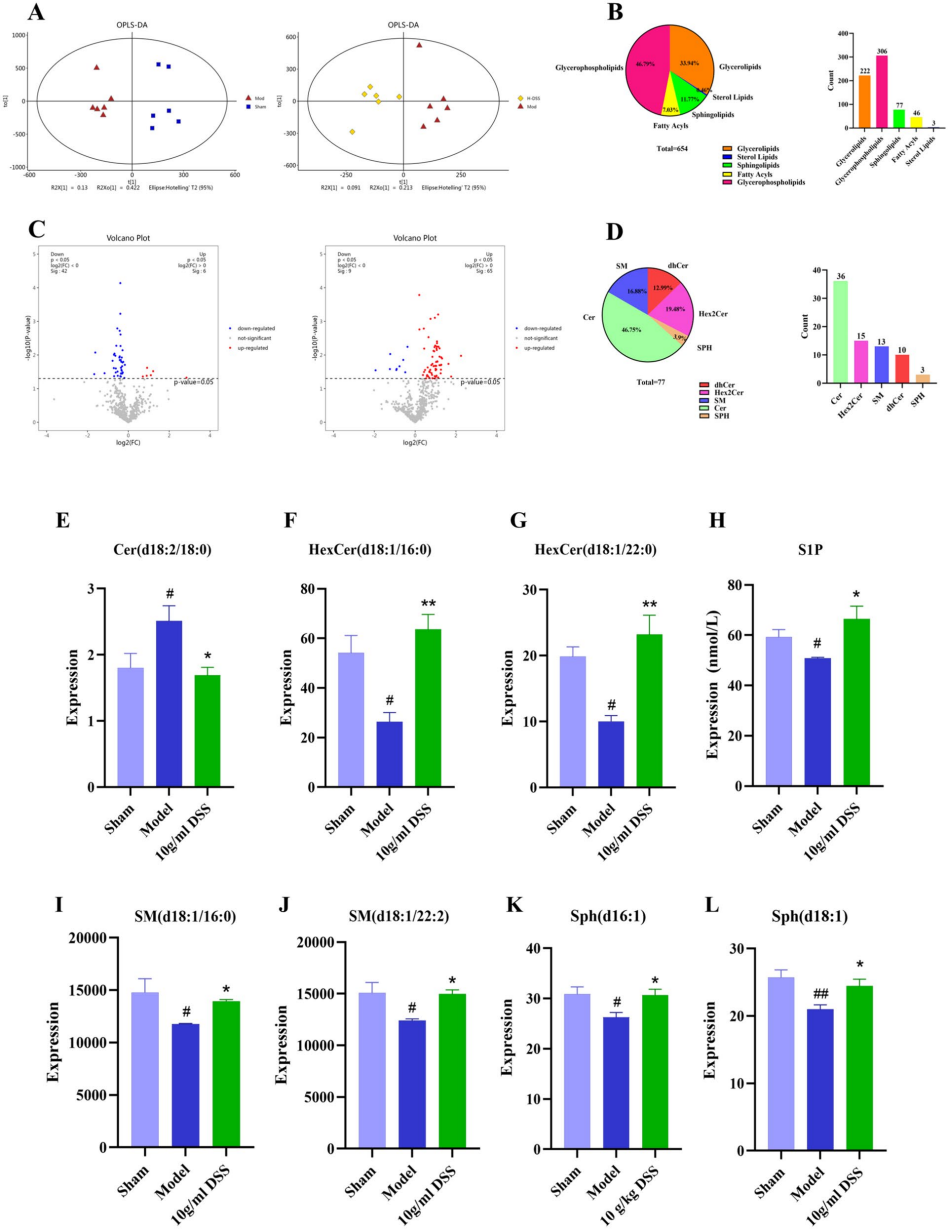
DSS improved sphingolipid metabolism levels in serum of VaD rats. **A** OPLS-DA score plots of lipid profiles in serum samples from three groups of rats. **B** The types and proportions of lipid metabolites in rat serum were detected using the LC–MS/MS method. **C** Volcano plot to visualize differential metabolites of significance between the sham/model group (left) and the model/H-DSS group (right). Metabolites with p value ≤ 0.05 are highlighted in red (up-regulated) and blue (down-regulated). **D** Types and ratios of lipid metabolites in sphingomyelin from rat serum. **E**-**L** Identification and quantification of sphingomyelins (Cer, SM, HerCer, S1P) between the three rat groups

Lipid metabolism, particularly that of sphingolipids, plays a crucial role in myelin regeneration. To investigate this, we compared the serum lipid profiles across different rat groups using targeted shotgun lipidomics analysis. OPLS-DA analysis revealed distinct separation trends between the sham and model groups, as well as between the model and H-DSS groups, with samples primarily clustering within the 95% confidence interval (Fig. 4A***&B).*** Utilizing LC–MS/MS, we detected a total of 654 lipid metabolites spanning five major subclasses (Fig. 4C&D). Among these, glycerophospholipids constituted the largest proportion (46.79%), followed by glycerides (33.94%) and sphingolipids (11.77%). Comparative analysis identified 48 significantly differential metabolites in the model group versus the sham group, of which 42 were down-regulated and 6 were up-regulated. Similarly, 73 differential metabolites were found in the H-DSS group compared to the model group, with 65 up-regulated and 9 down-regulated (Fig. 4E&F). Focusing on sphingolipids, a total of 77 metabolites were detected (Fig. 4G&H). Within this class, ceramides (Cer) represented the most abundant species (36). Compared to the sham group, the Cer (d18:2/22:2) level was significantly elevated in the model group (*P* < 0.05). Conversely, HerCer (d18:1/16:0), HerCer (d18:1/22:0), SM (d18:1/16:0), SM (d18:1/22:2), Sph (d18:1), Sph (d16:1) and S1P levels were significantly reduced (*P* < 0.05 or *P* < 0.01). Notably, DSS administration reversed these alterations in metabolite levels (*P* < 0.05) (Fig. 4I-P). Collectively, these results indicate that DSS can improve ameliorate serum sphingolipid metabolic profiles in VaD rats.

**Fig. 5:**
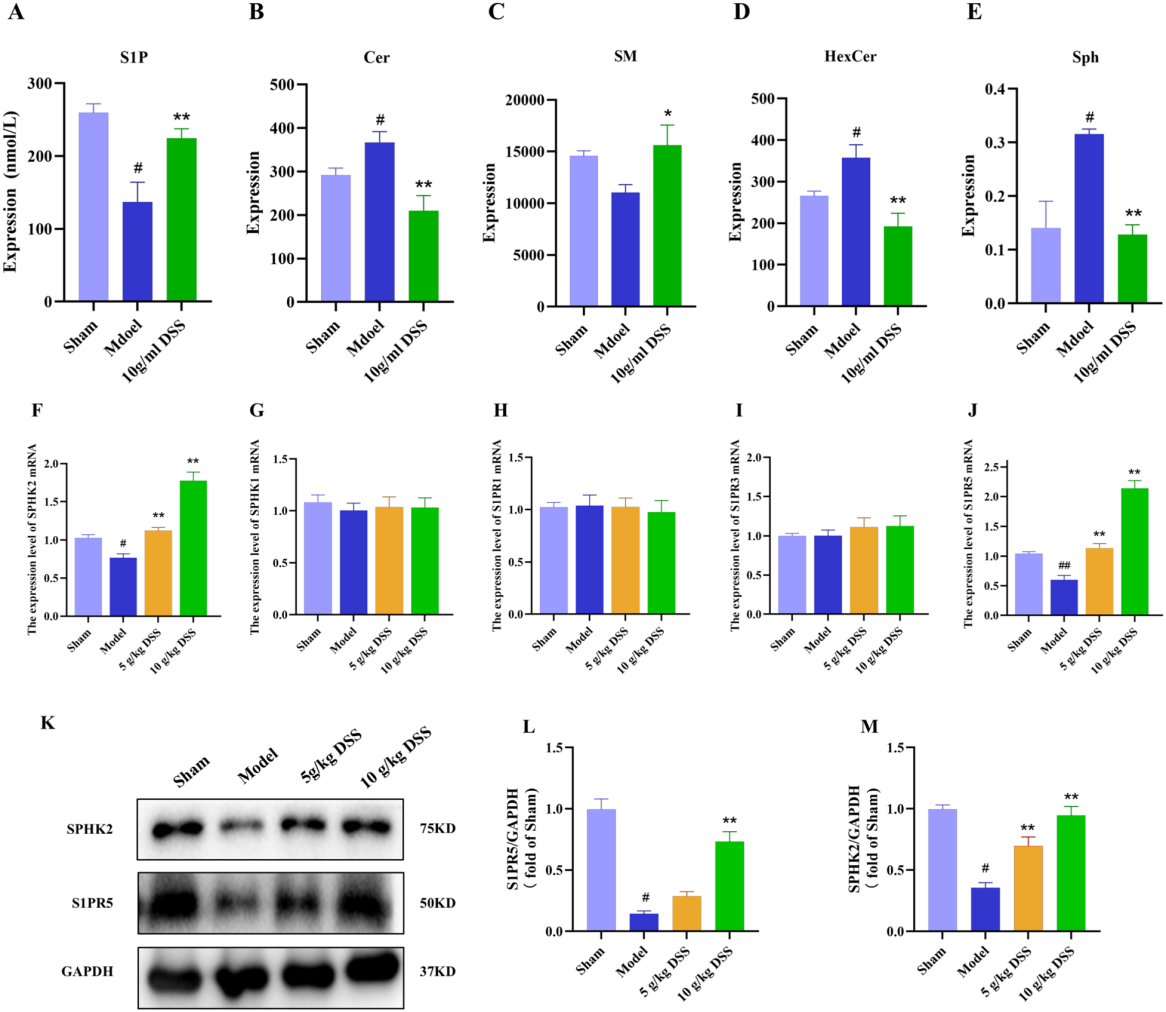
DSS activated the SPHK2/S1P/S1PR5 signaling in white matter of VaD rats. **A**–**E** Quantification of sphingolipid metabolites (S1P, Cer, SM, HexCer, Sph) in white matter using biochemical assays. **F**–**J** Gene expression analysis of sphingolipid metabolism enzymes and receptors (SPHK2, SPHK1, S1PR1, S1PR3, S1PR5). **K**–**M** Western blot analysis of sphingolipid-related proteins (SPHK2 and S1PR5)

We also quantified key metabolite levels of sphingolipid metabolites in rat white matter using biochemical kits and LC–MS/MS. As shown in Fig. 5A-E, compared to the Sham group, the model group exhibited significantly decreased levels of S1P and SM (*P* < 0.001), alongside significantly increased levels of Cer, HexCer, and Sph in the white matter. DSS significantly elevated SM and S1P levels (*P* < 0.01), and reduced Cer, HerCer, and Sph levels (*P* < 0.01). These results suggest that DSS may improve demyelination in VaD rats, at least in part, by modulating sphingolipid metabolite levels in the white matter.

### DSS activated the SPHK2/S1P/S1PR5 signaling in white matter of VaD rats

To further elucidate the potential molecular mechanism underlying DSS-mediated improvement of sphingolipid metabolism, we investigated key enzymes and signaling pathways in white matter. SPHK2, along with SPHK1, is a key enzymes catalyzing the phosphorylation of sphingosine to generate S1P. S1PR5, a specific receptor for S1P, is expressed in diverse cell types including neurons, glial cells and immune cells. Compared to the sham group, the model group exhibited significantly reduced mRNA levels of SPHK2 and S1PR5. DSS treatment upregulated SPHK2 and S1PR5 expression in VaD rats (Fig. 5F&J). In addition, we also examined the expression of SPHK1, S1PR1, and S1PR3, and found that DSS had no significant effect on the mRNA levels of these molecules (Fig. 5G-I). Western blot results corroborated the RT-qPCR findings. The protein expression of SPHK2 and S1PR5 in the model group was significantly reduced compared with that in the sham group (*P* < 0.0001). Conversely, H-DSS group exhibited markedly elevated levels of SPHK2 and S1PR5 compared to the model group (*P* < 0.001) (Fig. 5K-M). The above results suggest that SPHK2/S1P/S1PR5 may play a key role in the effect of DSS in modulating sphingolipid metabolism.

### SPHK2 inhibitor ABC294640 significantly diminished the improvement of DSS on cognitive impairment as well as sphingolipid metabolism in VaD rats

**Fig. 6:**
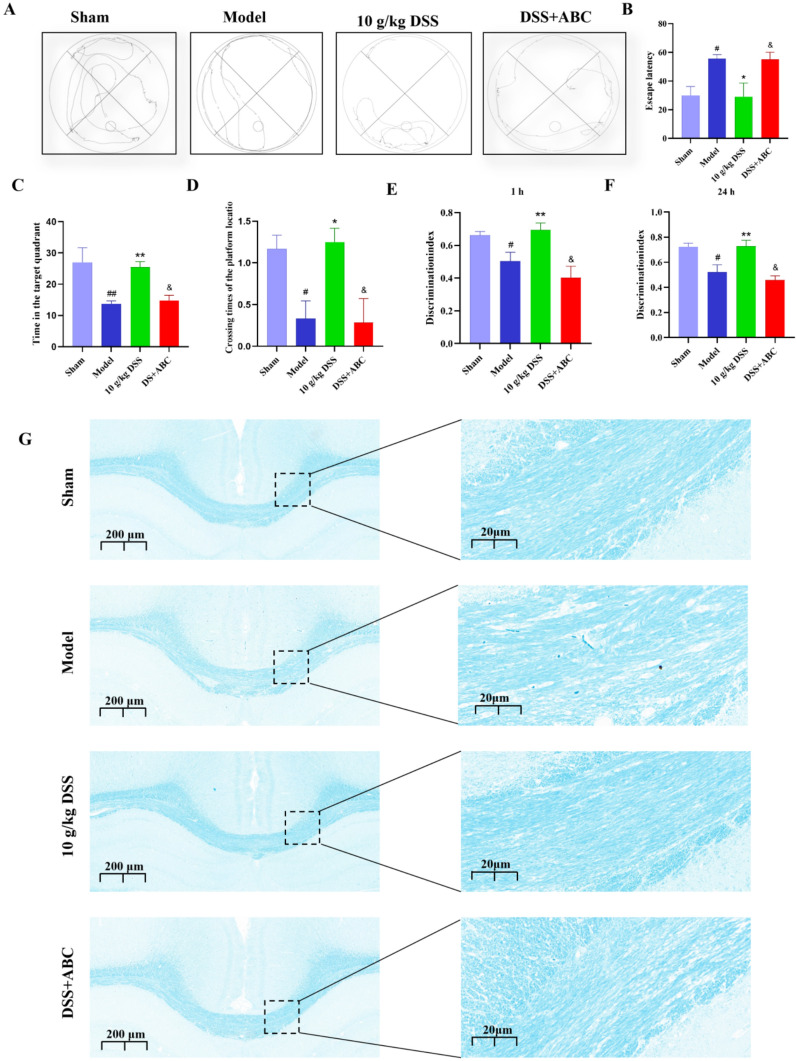
SPHK2 inhibitor ABC294640 significantly diminished the improvement of DSS on cognitive impairment and sphingolipid metabolism in VaD rats. **A**–**D** Morris water maze test performance after SPHK2 inhibition. **E**–**F** Novel object recognition test after SPHK2 inhibition. **G** Luxol Fast Blue (LFB) staining of white matter after SPHK2 inhibition

The above results suggest that DSS may exert its effects through activation of the SPHK2 pathway. However, the critical role of this pathway in the treatment of VaD still requires further verification. The present research further confirmed the contribution of SPHK2 in the efficacy of DSS using the SPHK2 inhibitor ABC294640. The water maze experiments revealed that, compared to DSS treatment alone, the combination of DSS with ABC294640 significantly decreased the target quadrant residence time, the number of platform crossings, while significantly increasing escape latency in rats (Fig. 6A-D). In novel object recognition tests, the cognitive index of rats was significantly lower in the model group versus the sham group (*P* < 0.05 and *P* < 0.01). DSS treatment significantly increased the cognitive index compared to the model group (*P* < 0.01). However, this improvement was significantly reduced in VaD rats receiving the combination of DSS and ABC294640 compared to DSS alone (Fig. 6E&F). Meanwhile, the results of LFB staining showed that co-administration of ABC294640 diminished the ameliorative effect of DSS on corpus callosum vacuolization, intensifying the vacuolization and impeding myelin sheath regeneration (Fig. 6G).

**Fig. 7:**
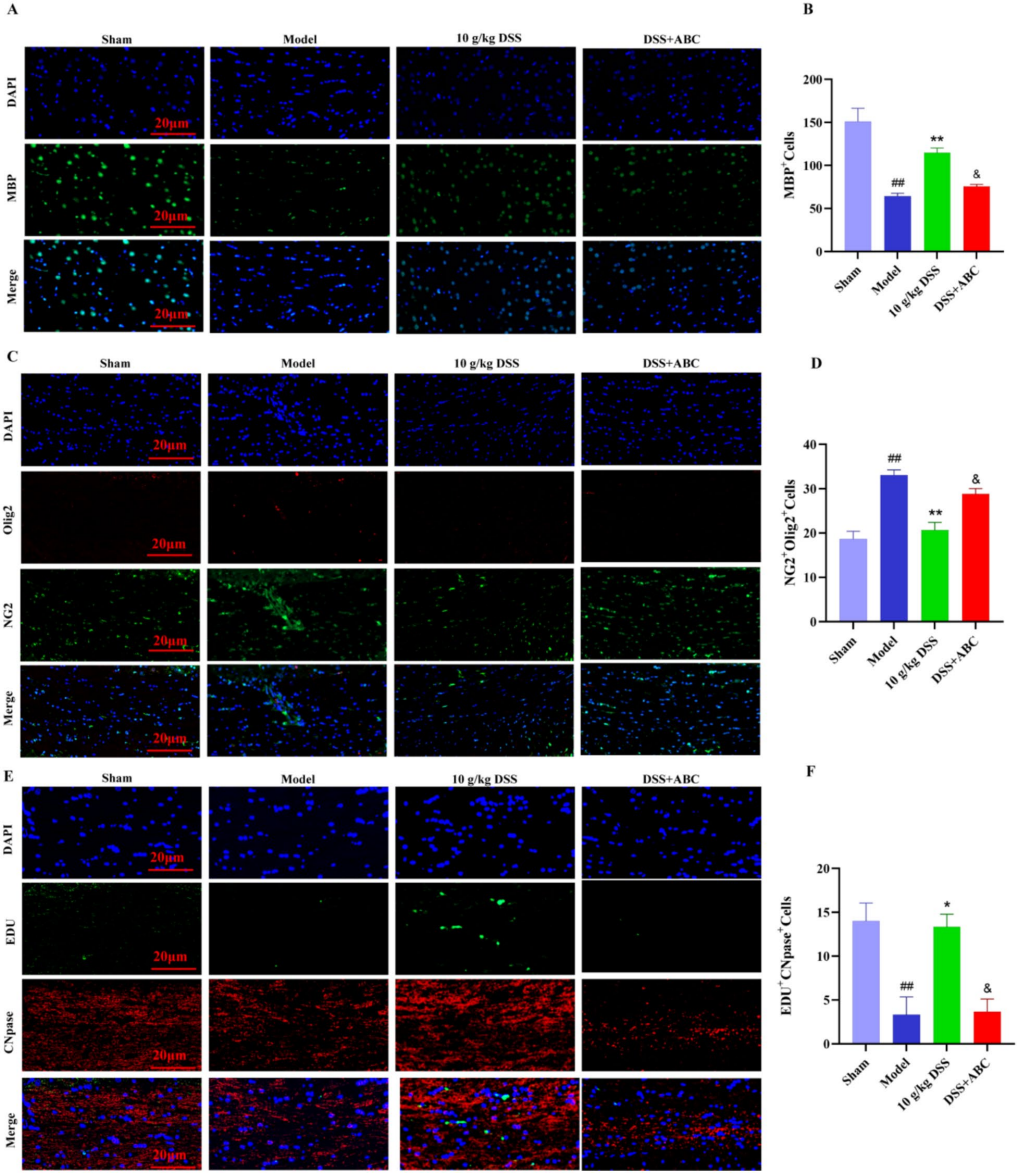
The effect of SPHK2 inhibitor ABC294640 on DSS-induced promotion of oligodendrocyte differentiation. **A** Immunofluorescence staining of MBP (myelin basic protein) in white matter tissue. **B** Quantification of MBP-positive cells from immunofluorescence staining. **C** Immunofluorescence staining of NG2, OLG2 in white matter tissue. **D** Quantification of NG2-positive cells from immunofluorescence staining. **E** Immunofluorescence staining of EdU and CNPase in white matter tissue. **F** Quantification of EdU-positive and CNPase-positive cells from immunofluorescence staining

Next, immunofluorescence was employed to further investigate the effect of ABC294640 on DSS-induced promotion of oligodendrocyte differentiation. MBP is one of the marker proteins for mature OLs. NG2^+^ cells mainly label OPCs, while Olig2^+^ cells can label the entire oligodendrocyte lineage, including OPCs and mature OLs. CNPase (2',3'-Cyclic Nucleotide 3'-Phosphodiesterase) is an enzyme that marks oligodendrocytes in the early stages of differentiation. EdU^+^ cells indicate cells that have undergone cell division (proliferation). Our experimental results show that, in the white matter tissue of VaD rats treated with DSS, the number of NG2^+^/Olig2^+^ cells was lower than that in the sham-operated group, while the number of MBP^+^ cells and EdU^+^/CNPase^+^ cells was significantly increased (Fig. 7A-F). These results suggested that the number of OPCs was significantly decreased whereas the number of mature OLs was significantly increased in the DSS-treated group. In contrast, in the ABC294640-treated group, the number of OPCs in the corpus callosum region was significantly increased and the number of mature OLs was decreased, suggesting that the SPHK2 inhibitor ABC294640 inhibited DSS-induced differentiation of OPCs into mature OLs.

These results suggest that ABC294640, an inhibitor of SPHK2, impaired the therapeutic effect of DSS on cognitive impairment in VaD rats. DSS likely promotes cognitive recovery by activating the SPHK2 signaling pathway to regulate sphingolipid metabolism.

### DSS improves cognition in VaD rats independently of gut flora

**Fig. 8:**
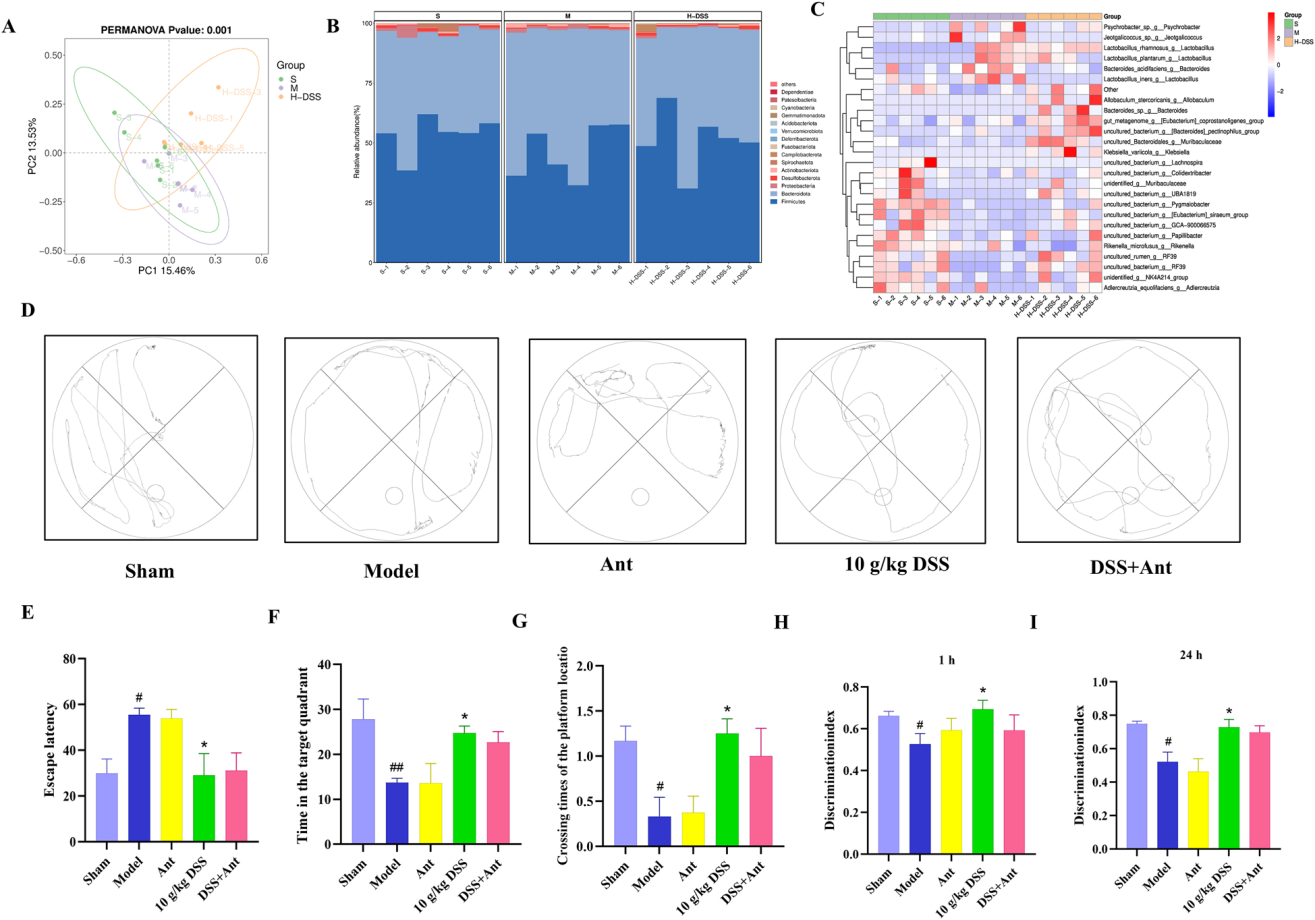
Effect of DSS on gut microbiota and cognitive function in VaD rats. **A** Principal Coordinate Analysis (PCoA) of gut microbiota composition from 16S rRNA gene sequencing, showing separation of microbial communities among the groups. **B** Relative abundance of gut microbiota at the phylum level across the groups. **C** The proportions of the main phyla, including Firmicutes, Bacteroidota, and others, in each group. **D** Path tracing of rats in the Morris water maze test across the groups. **E** Escape latency in the Morris water maze test across the groups. **F** Time spent in the target quadrant during the Morris water maze test across the groups. **G** Number of platform crossings in the Morris water maze test across the groups. **H** Cognitive index in the novel object recognition test at 1 h across the groups. **I** Cognitive index in the novel object recognition test at 24 h across the groups

Sphingolipid metabolism may also be regulated by various systemic factors. Given studies suggesting an important role for gut microbiota in regulating sphingolipid metabolism and neurological function, we further explored the effect of DSS on gut microecology. Using 16 s ribosomal RNA gene sequencing, we further investigated the potential effects of DSS on intestinal flora. Beta diversity analysis revealed significant the species variability among the samples, and principal coordinate analysis (PCoA) demonstrated clear separation of the microbial communities in the Sham, Model, and DSS groups (Fig. 8A). Analysis of relative abundance at the phylum level identified Firmicutes and Bacteroidota as the dominant phyla across all groups, followed by Proteobacteria, Desulfobacterota, Actinobacteria, and others (Fig. 8B & C). Among them, Bacteroidota are considered beneficial and are closely related to sphingolipid metabolism and nervous system development in vivo [26]. The current study confirmed that Bacteroidota can synthesize sphingolipids, such as sphingomyelin, dihydroceramide (DHCer) and ceramide phosphoethanolamine (CerPE). Importantly, compared to the model group, the DSS group exhibited a significant increase in the relative abundance of Bacteroidota.

Subsequently, we investigated whether DSS could improve cognitive function in VaD rats by modulating the gut microbiota through antibiotic cocktail-mediated gut flora depletion. The water maze results indicated that rats in the DSS group exhibited lower escape latency, as well as significantly higher residence time and number of traversals in the target quadrant compared to the model group. However, after depleting the intestinal flora of rats using an antibiotic combination, the escape latency, target quadrant residence time, and number of traversals in the DSS + Ant group were not significantly different from those in the DSS group (Fig. 8 D-G). In the novel object recognition test, the cognitive index of the model group was significantly lower than that of the sham group, while the DSS group showed a significantly higher cognitive index compared to the model group. The cognitive index of the DSS + Ant group was lower but not significantly different from that of the DSS group (Fig. 8H&I). Collectively, these findings suggest that depletion of intestinal flora does not attenuate the therapeutic effects of DSS on VaD, indicating that the neuroprotective effects of DSS are likely not mediated through the intestinal flora.

### Molecular docking, molecular dynamics simulation and MST validation

**Fig. 9:**
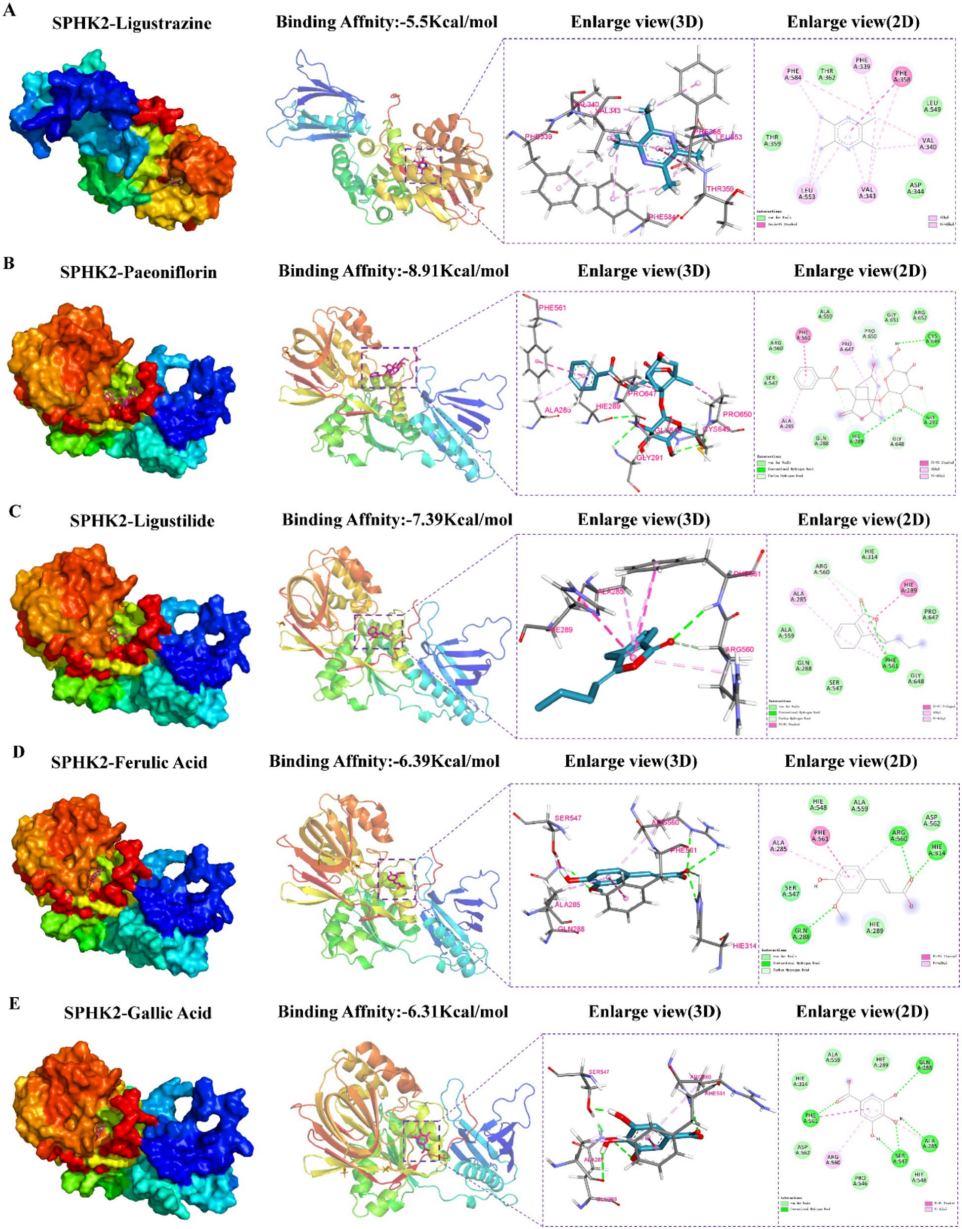
Molecular docking analysis of DSS active components with SPHK2. **A**–**E** The docking poses of ligustrazine (− 5.5 kcal/mol), albiflorin (− 8.91 kcal/mol), ligustilide (− 7.39 kcal/mol), ferulic Acid (− 6.39 kcal/mol), and gallic acid (− 6.31 kcal/mol) with SPHK2

After confirming the role of DSS in activating the SPHK2/S1P/S1PR5/SOX10 pathway, we further explored the interaction of DSS active components with SPHK2 by molecular docking analysis. Five major bioactive components, including ligustrazine, albiflorin, ligustilide, ferulic acid and gallic acid, were selected for molecular docking analysis and molecular dynamics simulation. As shown in Fig. 9, the above active components of DSS exhibited good spatial and energetic alignment with SPHK2. Lower MAT binding energy values were observed for Ligustrazine (− 5.5 kcal/mol), Albiflorin (− 8.91 kcal/mol), Ligustilide (− 7.39 kcal/mol), Ferulic acid (− 6.39 kcal/mol), and Gallic acid (− 6.31 kcal/mol), suggesting a strong affinity, especially for albiflorin and ligustilide, which may be the active ingredients of DSS that interact most strongly with SPHK2.

**Fig. 10:**
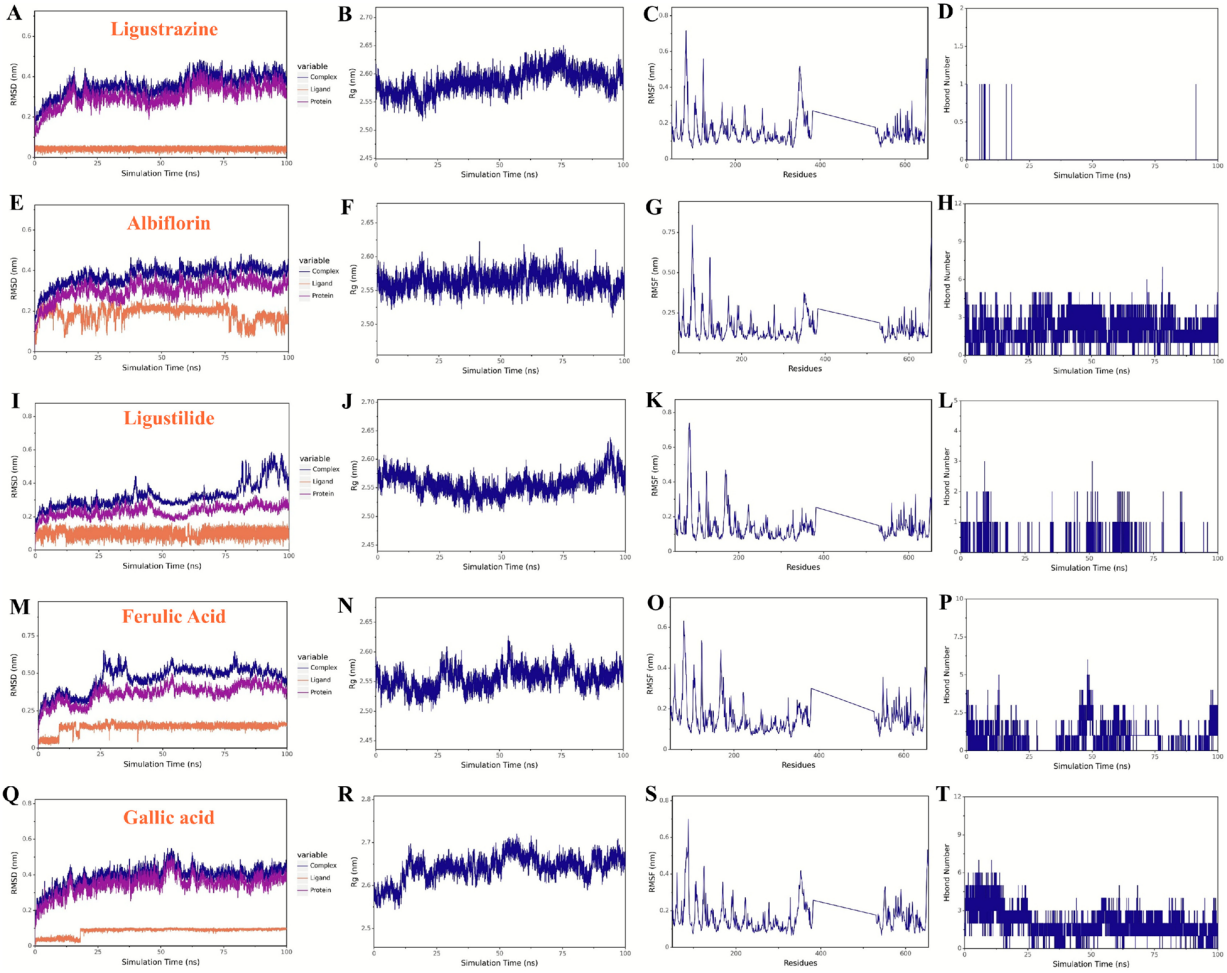
Molecular dynamics simulation results of DSS active components with SPHK2. **A**-**D** RMSD, Rg, RMSF and hydrogen bond number analysis for Ligustrazine-SPHK2 complex. **E**–**H** RMSD, Rg, RMSF and hydrogen bond number analysis for Albiflorin-SPHK2 complex. **I**-**L** RMSD, Rg, RMSF and hydrogen bond number analysis for Ligustilide-SPHK2 complex. **M**-**P** RMSD, Rg, RMSF and hydrogen bond number analysis for Ferulic Acid-SPHK2 complex. **Q**–**T** RMSD, Rg, RMSF and hydrogen bond number analysis for Gallic acid-SPHK2 complex

Root mean square deviation (RMSD) analysis was used to evaluate the structural stability of the ligand-SPHK2 complexes (Fig. 10 A, E, I, M, Q). The complexes of ligustrazine, albiflorin, and gallic acid demonstrated high stability, as their RMSD values rapidly converged to low plateaus. In contrast, the ligustilide-SPHK2 complex was stable initially but exhibited significant fluctuations after 80 ns, while the ferulic acid-SPHK2 complex showed marked instability throughout the simulation, indicative of transient binding. Collectively, these results indicate that ligustrazine, albiflorin, and gallic acid form the most stable complexes with SPHK2. In addition to root mean square deviation (RMSD) analysis, the radius of gyration (Rg) was calculated to evaluate the structural compactness of each ligand-SPHK2 complex during the simulation. The Rg analysis yielded trends consistent with the RMSD results: the Rg values of the ligustrazine, albiflorin, and gallic acid complexes gradually stabilized at low levels, indicating increased structural compactness. The ligustilide complex remained stable from 0 to 80 ns but exhibited fluctuations thereafter; whereas the ferulic acid complex showed frequent and significant Rg fluctuations throughout the simulation, further confirming its loose binding and poor stability (Fig. 10 B, F, J, N, R).

Root mean square fluctuation (RMSF) analysis further elucidated differences in binding stability. When bound to albiflorin and gallic acid, the protein residues exhibited lower RMSF values, indicating a more rigid and stable conformation. In contrast, complexes with ligustrazine, ligustilide, and ferulic acid showed higher RMSF values, suggesting greater flexibility in the binding regions and weaker stability (Fig. 10 C, G, K, O, S). Furthermore, hydrogen bond analysis revealed that albiflorin and gallic acid formed a greater number of hydrogen bonds with SPHK2 (ranging from 0–5 and 0–6, respectively), indicating stronger and more stable interactions. In comparison, the number of hydrogen bonds formed by ligustrazine, ligustilide, and ferulic acid fluctuated between only 0 and 1, reflecting weaker binding (Fig. 10 D, H, L, P, T). In summary, Rg, RMSF, and hydrogen bond analyses collectively demonstrate that albiflorin and gallic acid form more stable and structurally compact complexes with SPHK2, whereas the other three ligands exhibit relatively weaker binding stability.

**Fig. 11:**
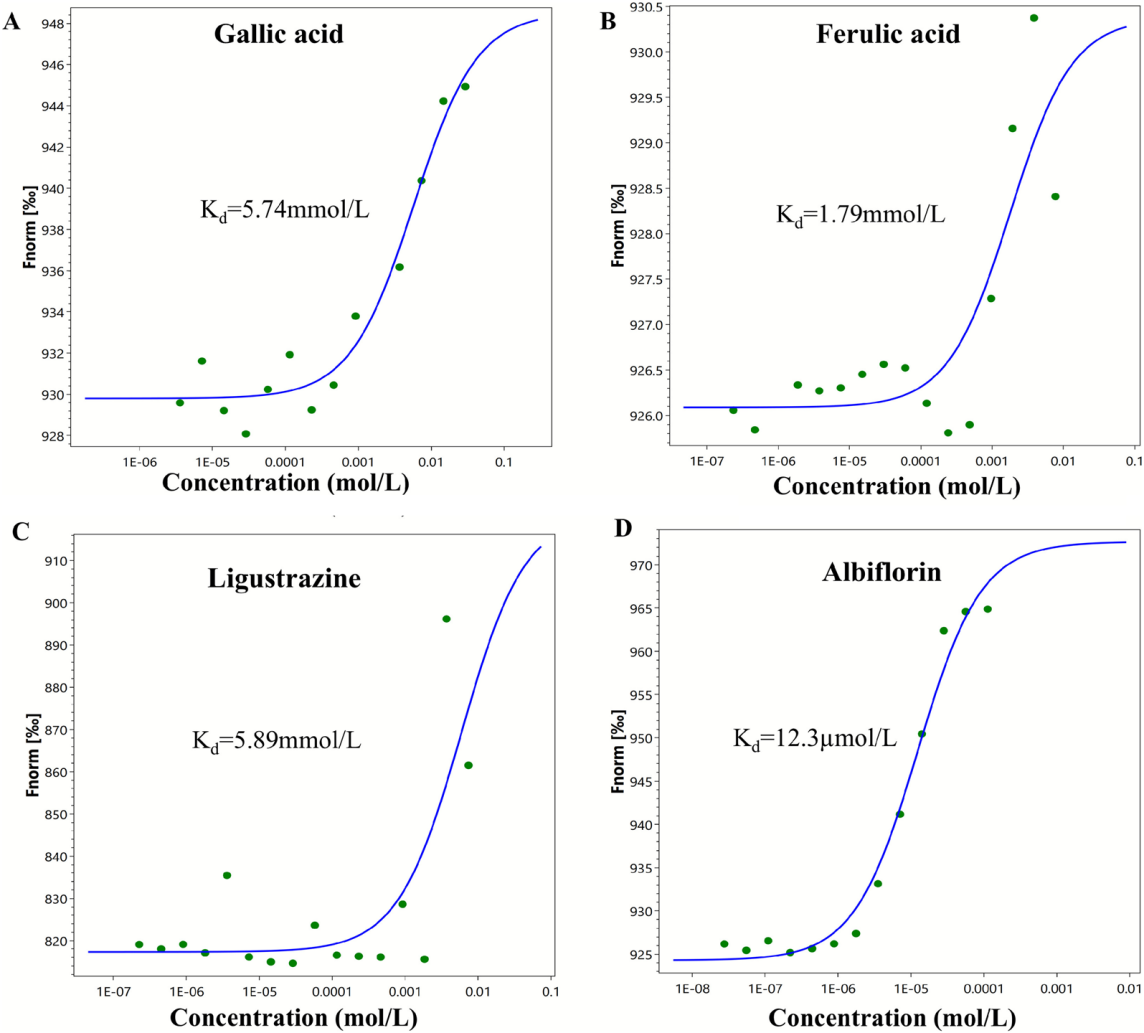
Microscale Thermophoresis (MST) experiment. **A**–**D** The binding affinities of Gallic acid (A), Ferulic acid (**B**), Ligustrazine (**C**), and Albiflorin (**D**) to SPHK2 protein were assessed using MST

Finally, microscale thermophoresis (MST) was employed to quantify the binding affinities of four compounds to SPHK2 (Fig. 11). Albiflorin exhibited the strongest binding with a Kd of 12.3 μM. In contrast, the other three compounds bound much more weakly: ferulic acid (Kd = 1.79 mM), gallic acid (Kd = 5.74 mM), and ligustrazine (Kd = 5.89 mM). These results clearly identify albiflorin as the most potent binder to SPHK2.

## Discussion

VaD is a disease strongly associated with cerebrovascular lesions, can induce cognitive impairment through disruption of myelin integrity [27, 28]. DSS, a traditional Chinese medicinal preparation, has demonstrated efficacy in improving cerebral blood flow to alleviate VaD symptoms, and exhibits neuroprotective effects in ex vivo studies [21, 29]. Our study confirms that DSS was able to significantly improve cognitive function in VaD rats. After DSS intervention, VaD rats showed marked improvement in the cognitive index of new object recognition, alongside a significant reduction in the escape latency, and a significant increase in the number of platform crossings in the Morris water maze. Concomitantly, LFB staining and TEM observations showed that DSS was able to repair myelin damage in the white matter region of VaD rats, restored myelin structure and reduced vacuolization. These results suggest that DSS successfully alleviated cognitive impairment in VaD rats by improving the integrity and function of myelin, proposing DSS as a potential drug for the treatment of VaD. Lipid metabolomics and WB results indicated that DSS exerted neuroprotective effects by activating the SPHK2/S1P/SOX10 pathway, promoting sphingolipid metabolism, and increasing S1P levels. Co-administration of SPHK2 inhibitor ABC294640 significantly diminishes the improvement of DSS on cognitive impairment as well as sphingolipid metabolism in VaD rats. Furthermore, gut microbiota analysis and antibiotic depletion experiments further confirmed that DSS regulates sphingolipid metabolism independently of gut flora. Molecular docking and molecular dynamics simulations indicated that the DSS components Albiflorin and Gallic acid form tighter and more stable interactions with SPHK2.

Myelin, a major component of white matter, is critical for efficient nerve signaling [30, 31]. Myelin damage and loss are important pathologic changes in the progression of VaD [32, 33]. Prolonged cerebral hypoperfusion leads to demyelination, structural disorganization of the corpus callosum, and disruption of white matter fiber bundles, thus ultimately impairing cognitive function [34]. In recent years, accumulating evidence underscores the importance of myelin protection and repair in VaD treatment strategies [35]. Sphingolipids are major components of both oligodendrocytes and myelin [36]. Perturbations in sphingolipid metabolism and its bioactive products can promote inflammation and contribute to VaD pathogenesis through multiple mechanisms, while also destabilizing myelin structures [37]. For instance, Simon et al. demonstrated that an imbalance between Cer and S1P significantly influences neuronal survival/death, and the proliferation/migration of neurons and vascular cells [38]. In our study, lipidomics experiments indicated that SM (d18:1/16:0), SM (d18:1/22:2), Sph (d18:1), Sph (d16:1) and S1P levels were significantly reduced, while Cer was accumulated in the white matter of VaD rats. This is consistent with the prior evidence linking sphingolipid metabolic imbalance to myelin damage [39]. In the VaD rat model, after DSS intervention, the accumulation of Cer was effectively suppressed, the level of S1P was significantly increased, and the Cer/S1P ratio was normalized. Further analysis revealed that DSS promoted myelin repair and improved cognitive function in VaD rats by regulating sphingolipid metabolism, particularly through restoring the dynamic balance of Cer/S1P.

Previous studies have established the critical role of sphingolipid metabolism in neuroprotection, particularly highlighting the impact of the dynamic balance between Cer and S1P metabolism on VaD. Further investigations reveal that the synthesis of S1P is tightly regulated by key enzymes, primarily members of the sphingosine kinase (SPHK) family, including SPHK1, SPHK2, and SPHK5 [40, 41]. In previous studies, the role of SPHK1 has been extensively studied, particularly in relation to neuroprotection and neurodegenerative diseases [42, 43] SPHK1 is predominantly expressed in most tissues, where it regulates cell growth and survival. In contrast, SPHK2 is primarily localized within the central nervous system (CNS), particularly in oligodendrocytes and neurons, where it plays a key role in mediating neuroprotective responses. SPHK5, on the other hand, functions in brain cells and cardiac tissue to regulate cell migration and myelin formation [44]. Collectively, these enzymes coordinate essential nervous system functions by catalyzing the conversion of sphingosine to sphingosine-1-phosphate (S1P). In our study, we demonstrated that DSS promotes the production of S1P through the activation of SPHK2, which is mainly localized in the nucleus and oligodendrocytes of the CNS. This suggests that, although SPHK1 plays a critical role in various tissues, SPHK2 may be more pivotal in the context of VaD, particularly in the repair of myelin and the restoration of cognitive function. S1P generated by SPHK2 acts through its receptor S1PR5 to further activate the transcription factor SOX10, which promotes the differentiation of OPCs to mature OLs and enhances myelin regeneration. Our experimental results indicate that DSS significantly promotes the differentiation of OPCs, ameliorates myelin damage, and restores cognitive function by activating the SPHK2/S1P/SOX10 pathway. In addition, sphingolipid metabolism played a key role in this process. Sphingolipid production catalyzed by SPHK2 was an important part of sphingolipid metabolism, and S1P played an important role in neuroprotection [45]. Intervention with the SPHK2 inhibitor ABC294640 significantly attenuated the improvement effect of DSS on cognitive improvement. This finding confirms that the SPHK2/S1P/SOX10 pathway is a key mechanism by which DSS exerts neuroprotective effects.

Recent studies have highlighted the influence of gut microbiota on host sphingolipid metabolism [46]. Our sequencing analyses identified DSS was able to alter the composition of the intestinal microbiota, especially increasing the proportion of Bacteroidota, a phylum associated with sphingolipid metabolism and nervous system development [47, 48]. Nevertheless, despite the potential role of gut microbiota in sphingolipid metabolism, the current study found no evidence for a direct impact of gut microbiota on S1P synthesis. Crucially, the brain remains the primary source of S1P. Remarkably, antibiotic depletion experiments further revealed that DSS-mediated cognitive improvement in VaD rats was not attributable to gut microbiota modulation. Thus, despite the ability of DSS to modulate the composition of the gut flora, the neuroprotective effect of DSS primarily stem from the direct regulation of sphingolipid metabolism within the CNS. Specifically, DSS activates the SPHK2/S1P/SOX10 pathway to promote OPCs differentiation into mature OLs and enhance myelin regeneration.

Although our study elucidated the potential mechanisms of DSS in VaD treatment, some limitations remain. First, we did not employ SPHK2-knockout mouse models to investigate the role of DSS under conditions of specific gene knockout or overexpression. Utilizing transgenic models could provide more precise insights into the contribution of SPHK2/S1P/SOX10 signaling pathway in VaD therapy. Second, while we presented robust evidence from rat models, the lack of in vitro cellular validation and lentiviral transduction experiments limits our exploration of DSS’s mechanism of action at the cellular level. Finally, there are still other potential mechanisms that may play a role in the neuroprotective effects of DSS have not been fully explored in this study. Future research may further explore the effects of other signaling pathways and cellular processes on the efficacy of DSS, thus enhancing our understanding of its multifaceted mechanisms of action.

In summary, the current study demonstrates that DSS significantly improves cognitive function in VaD rats by promoting OPC differentiation and myelin regeneration. This effect is achieved through activation of the SPHK2/S1P/S1PR5/SOX10 signaling pathway. Our findings provide novel theoretical foundation supporting DSS as a potential therapeutic agent for VaD.

## Data Availability

No datasets were generated or analysed during the current study.

## Abbreviations

AD: Alzheimer’s Disease
Cer: Ceramide
CerPE: Ceramide phosphoethanolamine
DHCer: Dihydroceramide
DSS: Danggui Shaoyao San
ELISA: Enzyme-linked immunosorbent assay
LC–MS/MS: Liquid chromatography-tandem mass spectrometry
LFB: Luxol Fast Blue
MAG: Myelin-associated glycoprotein
MAPK: Mitogen-activated protein kinase
MBP: Myelin basic protein
MD: Molecular dynamics
Myrf: Myelin regulatory factor
NIM: Nimodipine
Ols: Oligodendrocytes
OPCs: Oligodendrocyte precursor cells
OPLS-DA: Orthogonal partial least squares-discriminant analysis
PCoA: Principal coordinate analysis
Rg: Radius of gyration
RMSD: Root mean square deviation
RMSF: Root mean square fluctuation
RT-qPCR: Real-time quantitative PCR
SPHK: Sphingosine kinase
S1P: Sphingosine-1-phosphate
Sph: Sphingosine
S1PR5: Sphingosine-1-phosphate receptor 5
SOX10: SRY-box transcription factor 10
2VO: The bilateral common carotid artery ligation method
TEM: Transmission electron microscopy
VaD: Vascular dementia
